# The Impact of a Cognitive–Behavioral Therapy on Event-Related Potentials in Patients with Tic Disorders or Body-Focused Repetitive Behaviors

**DOI:** 10.3389/fpsyt.2016.00081

**Published:** 2016-05-10

**Authors:** Simon Morand-Beaulieu, Kieron P. O’Connor, Maxime Richard, Geneviève Sauvé, Julie B. Leclerc, Pierre J. Blanchet, Marc E. Lavoie

**Affiliations:** ^1^Laboratoire de psychophysiologie cognitive et sociale, Montreal, QC, Canada; ^2^Centre de recherche de l’Institut universitaire en santé mentale de Montréal, Montreal, QC, Canada; ^3^Département de neurosciences, Faculté de médecine, Université de Montréal, Montreal, QC, Canada; ^4^Département de psychiatrie, Faculté de médecine, Université de Montréal, Montreal, QC, Canada; ^5^Département de psychologie, Faculté des sciences humaines, Université du Québec à Montréal, Montreal, QC, Canada; ^6^Département de stomatologie, Faculté de médecine dentaire, Université de Montréal, Montreal, QC, Canada

**Keywords:** Tourette syndrome, tic disorders, body-focused repetitive behaviors, habit disorder, cognitive–behavioral therapy, cognitive–psychophysiological therapy, event-related potentials, electrophysiology

## Abstract

**Context:**

Tic disorders (TD) are characterized by the presence of non-voluntary contractions of functionally related groups of skeletal muscles in one or multiple body parts. Patients with body-focused repetitive behaviors (BFRB) present frequent and repetitive behaviors, such as nail biting or hair pulling. TD and BFRB can be treated with a cognitive–behavioral therapy (CBT) that regulates the excessive amount of sensorimotor activation and muscular tension. Our CBT, which is called the cognitive–psychophysiological (CoPs) model, targets motor execution and inhibition, and it was reported to modify brain activity in TD. However, psychophysiological effects of therapy are still poorly understood in TD and BFRB patients. Our goals were to compare the event-related potentials (ERP) of TD and BFRB patients to control participants and to investigate the effects of the CoPs therapy on the P200, N200, and P300 components during a motor and a non-motor oddball task.

**Method:**

Event-related potential components were compared in 26 TD patients, 27 BFRB patients, and 27 control participants. ERP were obtained from 63 EEG electrodes during two oddball tasks. In the non-motor task, participants had to count rare stimuli. In the motor task, participants had to respond with a left and right button press for rare and frequent stimuli, respectively. ERP measures were recorded before and after therapy in both patient groups.

**Results:**

CoPs therapy improved symptoms similarly in both clinical groups. Before therapy, TD and BFRB patients had reduced P300 oddball effect during the non-motor task, in comparison with controls participants. An increase in the P300 oddball effect was observed posttherapy. This increase was distributed over the whole cortex in BFRB patients, but localized in the parietal area in TD patients.

**Discussion:**

These results suggest a modification of neural processes following CoPs therapy in TD and BFRB patients. CoPs therapy seems to impact patients’ attentional processes and context updating capacities in working memory (i.e., P300 component). Our results are consistent with a possible role of the prefrontal cortex and corpus callosum in mediating interhemispheric interference in TD.

## Introduction

Tic disorders (TD) are characterized by repetitive non-voluntary contractions of functionally related groups of skeletal muscles in one or more parts of the body, including blinking, cheek twitches, and head or knee jerks among others. Tics can also be more complex and take the form of self-inflicted repetitive actions, such as teeth grinding, head slapping, or tense-release hand gripping cycles. They also appear as more purposive and stereotyped movements of longer duration, such as facial gestures and grooming-like movements. Furthermore, tics can be vocal, and they range from simple sounds, such as sniffing, coughing, or barking, to more complex vocalizations, such as echolalia or coprolalia. The tics may wax and wane over the course of weeks, months, and years. They can appear in bouts many times a day with onset longer than a year and arise prior to 18 years old with a peak in symptoms intensity around 12 years old. Tourette syndrome, which is the best known TD, involves multiple motor tics and at least one vocal tic. In comparison, persistent TD implies either motor or phonic tics, but not both. Tourette syndrome and persistent TD patients are often pooled together as a sole group, and the need for a distinction between both has been debated, since phonic tics have an inherent motor component (1).

Recent brain imaging investigations have revealed impairment in cortico-striato-thalamo-cortical (CSTC) pathways, which assure the communication between the basal ganglia and the motor cortex (2–4). At the cortical level, the overactivity of the supplementary motor area (SMA) was also observed in TD. The SMA is an important structure related, in large part, to the generation of tics and also to sensory urges (5, 6). Consistent with these findings, gray matter thinning was also found within the SMA, and this was also correlated to the severity of tics (7) and premonitory urges (8).

The large majority of patients with TD also face various comorbidities (9), which include obsessive–compulsive disorder (OCD) or at least some obsessive–compulsive symptoms (OCS), attention-deficit hyperactivity disorder (ADHD), depression, and anxiety disorders. Another pathology often associated with TD is body-focused repetitive behaviors (BFRB), also known as habit disorder. BFRB represent a clinical term that includes various diagnoses, such as trichotillomania, skin picking, and onychophagia. Despite the heterogeneity of symptoms comprised of the BFRB category, their main symptoms are directed toward the body, in reaction to feelings of discomfort, which is often present in TD. In the DSM-IV-TR, trichotillomania was categorized as an impulse control disorder, not elsewhere classified, and was associated with skin picking and onychophagia (10). In the DSM-V, trichotillomania and skin picking are now classified within the obsessive–compulsive and related disorders category, while onychophagia and dermatophagia are mentioned as “other specified obsessive–compulsive and related disorders.” Despite the fact that these disorders have been relocated to the obsessive–compulsive category, impulse control and feeling of sensory discomfort remain an important communality of their profile. This incapacity to resist a specific impulse or urge is a characteristic shared with TD patients. Both groups also show heightened levels of sensorimotor activation (11–13). However, even though BFRB resemble to TD in certain ways and these two disorders sometimes co-occur with one another, it must be noted that are different diagnoses.

There is a clear benefit in distinguishing between TD and BFRB, for the reason that the relationship between these two entities is sometimes clinically unclear, because the presence of complex movements in BFRB can often be confounded with complex tics. We propose that a reasonable method of differentiating these two groups would be to compare directly their brain activity during the performance of contrasting tasks with different levels of motor demand. For instance, O’Connor et al. (14) reported that TD and BFRB patients both failed to adequately adjust their hand responses to automated or controlled movements. More precisely, TD patients had the most severe impairment in synchronizing motor-related brain activity with their actual response time, followed by the BFRB and the control groups. These findings give support to a dimensional model of classification with BFRB falling between TD and controls along a continuum of motor arousal.

Recent brain imaging investigations on trichotillomania suggest that BFRB could share common impaired neural networks with TD, affecting mainly motor processing. For instance, increased gray matter density in the left striatum, the left amygdalohippocampal formation, the cingulate gyrus, the SMA, and the frontal cortex was found in trichotillomania (15). Furthermore, BFRB patients with trichotillomania or skin picking as their main habit have less fractional anisotropy in the anterior cingulate and temporal areas, which indicate a lower fiber density, axonal diameter, and myelination in white matter tracts involved in motor habits generation and suppression (16, 17). Additional circuits seem affected in unmedicated TD, where engagement in habit formation behavior correlated with greater connectivity of motor structures in the right hemisphere and stronger structural connectivity between the SMA and the putamen, which predicted more severe tics (18). All in all, aberrant reinforcement signals to the sensorimotor cortex and the striatum might be crucial for habit formation and tic generation as well. These areas are all known to be involved in cognition and habit learning and could contribute to the development of pathological habits, but more research are needed to incorporate other types of impulse control disorders.

Another good reason to characterize TD and BFRB is mainly related to their response to treatment. Currently, cognitive–behavioral therapy (CBT) constitutes an effective line of treatment for adults with both TD (19, 20) and BFRB (21–24), but the cognitive–behavioral and physiological outcomes are not well understood. The therapy proposed by our group is based on the cognitive–psychophysiological (CoPs) model and aims at regulating the high level of sensorimotor activation present in these populations and preventing the build-up of tension that leads to tic bursts or to the compulsive habit related to BFRB (12, 25, 26). Its effectiveness in treating adults affected by either disorder has been demonstrated many times (26–28). The positive effects of the CoPs therapy in TD patients are also reflected at the cerebral level. This was first reported with a TD group, which showed reduced electrocortical activity related to the inhibition of automatic motor responses. It was shown that the motor-related brain response during automatic inhibition, normalized following successful CoPs therapy (29). These results are also consistent with fMRI recordings during a motor inhibition task, which found a significant decrease in putamen activation after cognitive–behavioral treatment in adult TD (30). More recently, the CoPs therapy induced a reduction of the lateralized readiness potentials, a brain electrical potential partly generated by the SMA and the basal ganglia (13). Thus, these results are strongly consistent with the cortical–striatal and basal ganglia impairment hypothesis in TD. More importantly, these results showed that psychological treatments have the potential to induce changes in behavior and cognitive processes that are followed by modification of brain activity. The next question to explore is the cerebral impact of therapy in the BFRB.

One effective way to follow various levels of cognitive and electrocortical activity within milliseconds accuracy is the use of event-related potentials (ERPs). Thus, we specifically aimed at the investigation of three ERP components, the P200, the N200, and the P300 recorded at pre- and posttherapy. The P200 is a component that indexes evaluation of stimulus salience and its task-related adequacy (31, 32). The N200 indexes target detection and conflict monitoring (33), whereas the P300 is related to stimulus evaluation and context updating in working memory (34). To the best of our knowledge, no study has, so far, investigated the ERPs in BFRB patients, although several have studied TD patients (35–42). Thus, our first goal is to compare specific ERP components in TD and BFRB patients before any treatment. Our second aim is to focus on cerebral changes that accompany behavioral and cognitive modification, after CoPs therapy. We expect an improvement in tics and habits symptoms in TD and BFRB patients, respectively. The main hypothesis predicts that TD and BFRB patients will show intact early evaluation of salience as reflected by the P200 (31, 32), while showing larger target detection and conflict monitoring as indexed by a larger N200 (33), which is consistent with earlier clinical findings with TD reporting an intact P200 amplitude (42), and larger N200 amplitude (39). Finally, we hypothesize a reduced P300 oddball effect in our clinical groups, which was also consistently found in TD patients with OCS (42), with OCD (43–46), and without comorbidity (39, 47). Such reduced P300 would indicate a decrease in memory updating processes (34) in both disorders. We propose to contrast ERPs across motor and non-motor oddball tasks, which will ascribe the contribution of motor responses. Earlier studies involving healthy participants with the counting and the motor oddball task showed activation of the SMA, the cerebellum, the thalamus, and the parietal cortex. However, activation of the middle frontal gyrus central opercular cortex and parietal operculum was specific to the motor oddball task, suggesting a specific contribution of these regions in action execution (48). Finally, we hypothesize an equivalent normalization of the P300 in both patient groups after treatment.

## Materials and Methods

### Participants

Patients with either TD or BFRB were recruited from the *Centre d’études sur les troubles obsessionnels-compulsifs et les tics* from the *Centre de recherche de l’Institut universitaire en santé mentale de Montréal* to participate in this study. Patients with TD as their main concern were assigned to the TD group. Therefore, the TD group was composed of 26 patients who met the DSM-IV-TR criteria for either Tourette syndrome (307.23) or chronic TD (307.22) (10). Patients with BFRB as their main concern were assigned to the BFRB group. The latter group was composed of 27 patients with specific habit disorders, such as trichotillomania (*n* = 12), onychophagia (*n* = 8), skin picking (*n* = 5), and bruxism (*n* = 2). These two patients’ groups were matched to a group of 27 healthy controls on the basis of age, intelligence (Raven), and laterality.^1^ The project was approved by the ethics committee of the *Centre de recherche de l’Institut universitaire en santé mentale de Montréal*, and all participants granted their written informed consent, in accordance with the Declaration of Helsinki. Seven TD patients and four BFRB patients were under medication during the study. Those medication were α2-adrenergic agonists (*n* = 1), β_2_-adrenergic agonists (*n* = 1), antidepressants (*n* = 7), benzodiazepine (*n* = 3), non-benzodiazepine (*n* = 1) hypnotics, neuroleptics (*n* = 2), and lithium (*n* = 1). However, to be included in our study, their medication had to remain stable throughout the entire process. Socio-demographic characteristics of our participants can be found in Table 1.

**Table 1:** **Socio-demographic and clinical characteristics**.

	TD (*n* = 26)		BFRB (*n* = 27)		Controls (*n* = 27)				
	Mean	SD	Mean	SD	Mean	SD	*F*	*p*	Group difference
Age	38	11.9	40	14.4	36	13.0	0.48	ns	
Sex (% of males)	65%	N/A	26%	N/A	41%	N/A	4.60*	**<0.05**	TD > BFRB
Intelligence (percentiles)	88	13.8	80	17.2	84	17.1	1.49	ns	
Laterality (R:L:A)	24:2:0	N/A	24:3:0	N/A	25:0:3	N/A	5.42^a^	ns	
OCS (Padua)	32	32.1	35	25.8	17	15.6	4.14*	**<0.05**	BFRB > controls
Depression (BDI)	11	10.2	14	7.8	3	3.8	15.70***	**<0.001**	TD and BFRB > controls
Anxiety (BAI)	8	5.9	11	6.6	5	4.6	7.19**	**<0.01**	BFRB > controls
Impulsivity (BIS-10)^b^	71	8.8	72	7.9	64	8.7	5.82**	**<0.01**	TD and BFRB > controls

*Laterality: R, right-handed; L, left-handed; A, ambidextrous. Intelligence: Raven’s matrices percentiles; OCS, obsessive–compulsive symptoms; BDI, Beck depression inventory; BAI, Beck anxiety inventory; BIS-10, Barratt impulsiveness scale; ns, not statistically significant*.

*p < 0.05.

**p < 0.01.

***p < 0.001.

*^a^Fisher’s exact test was used to analyze categorical data with cells containing an expected count below 5*.

*^b^One TD patient and eight controls with missing data*.

*Every significant result is in bold*.

Exclusion criteria consisted of the presence of a psychiatric diagnosis, such as schizophrenia, mood disorders, somatoform disorders, dissociative disorders, and substance-related disorders. The presence of personality disorders was screened with the personality diagnostic questionnaire-fourth edition (49–51), and participants with personality disorders were excluded. Other medical conditions, such as neurological diseases, were screened by a neurologist (Pierre J. Blanchet) and were also a criterion for exclusion.

### Procedures

#### Clinical Assessment

Patients underwent a battery of psychological tests to assess symptoms. The Tourette Syndrome Global Scale [TSGS (52)] and the Yale Global Tic Severity Scale [YGTSS (53)] were used to assess tics symptoms in TD patients. We adapted the TSGS and the YGTSS to assess the presence of habit disorders in the BFRB group. In these adapted versions of both questionnaires, the word “tic” was replaced by the word “habit.” These questionnaires were adapted to quantify both tics and habits on the same metric uniformly. This adaptation has been validated in a prior research from our group (54).

We also used the *Massachusetts General Hospital Hair Pulling Scale* [MGH-HPS (55)] to assess BFRB severity. The MGH-HPS is a seven-point inventory measuring the severity of trichotillomania symptoms. Again, an adaptation of this scale was proposed to assess onychophagia, skin picking, and skin scratching. Therefore, the current data reported in the MGH-scale column reflected the severity score of the principal habit of each BFRB patient. Good convergent validity was found between TSGS and MGH scales, as prior research found correlations between TSGS tic scores and the MGH-HPS (*r* = 0.49, *p* < 0.05), as well as the MGH scales adapted for nail biting and skin picking (*r* = 0.52, *p* < 0.05) (54).

Obsessive–compulsive symptoms were assessed with the Padua inventory (56). The 10th version of the Barratt Impulsiveness Scale (BIS-10) was administered to assess impulsivity in our participants (57). The Beck anxiety inventory [BAI (58)] and the Beck depression inventory [BDI (59)] were used to assess anxiety and depression symptomatology, respectively. The occurrence of anxiety disorders was assessed by a structured interview with the anxiety disorders interview schedule (60). Severe psychological stressors, time availability, and other psychological problems were also screened.

#### Cognitive–Behavioral Therapy Based on the Cognitive–Psychophysiological Model

The two clinical groups, which are composed of 26 patients with TD and 27 patients with BFRB, underwent the same CBT, based on the cognitive–psychophysiological (CoPs) model (12). This treatment, while including some classic principles of symptom awareness and habit reversal therapy, focuses on cognitive and behavioral restructuration in situations presenting a high risk for tic bouts. The therapy was delivered by two licensed psychologist (supervised by Kieron P. O’Connor) on a weekly one-to-one basis. The treatment program includes basic clinical steps, which are cumulative and administered over 14 60-min sessions: awareness training (psychoeducation, daily diary, video, situational profile), muscle discrimination (gradation of tension, normalize contractions), muscular relaxation, reducing sensorimotor activation, modifying background style of action, cognitive and behavioral restructuring (development of alternative goal driven responses using cognitive and behavioral strategies), generalization, and preventing relapse.^2^ At the end of the 14th week, there is a home-based practice period lasting 4 weeks with weekly phone contact with the therapist to ensure compliance and deal with trouble shooting. Therefore, there was a time lapse of 18 weeks between the beginning of the program and the posttreatment evaluation. Conditions of treatment delivery, duration, homework, and treatment monitoring were equivalent and supervised for integrity.

#### Oddball Paradigms

Two types of oddball paradigms were used in this study. During both oddball tasks, 200 black letters (X and O on a white background) were randomly presented during 100 ms on a computer screen (Viewsonic SVGA 17″ monitor), with a random 1700–2200 ms inter-trial interval. The frequent stimulus (the letter “O”) was presented 80% of the time (*n* = 160), whereas the rare stimulus (the letter “X”) was presented with a 20% probability (*n* = 40). The first task is a *counting oddball task*, which presented the same stimuli, but this time participants must only count the number of rare stimuli. At the end of the experiment, the participants had to report the exact amount of rare stimuli (*n* = 40). The second task is a *motor oddball task*, where participants pressed the keyboard left arrow key with their left index finger when frequent stimuli were presented and pressed the right arrow key with their right index finger, when the rare stimuli were presented. The order of presentation of the counting and the motor tasks was counterbalanced across participants.

### Electrophysiological Recordings

The EEG was recorded during both oddball tasks, with a digital amplifier (Sensorium Inc., Charlotte, VT, USA). EEG signal was recorded from 63 Ag/AgCl electrodes mounted in a lycra cap (Electrode Arrays, El Paso, TX, USA)^3^ and placed according to standard EEG guidelines (61). All electrodes were referenced to the nose. The signal was sampled continuously at 500 Hz and recorded with 0.01 Hz high-pass filter and a 100-Hz low-pass filter (60 Hz notch filter). Impedance was kept below 5 kΩ, using an electrolyte gel (JNetDirect Biosciences, Herndon, VA, USA). Bipolar electro-oculogram (EOG) was recorded to clear EEG from eye artifacts, such as blinks and eye movements. Electrodes were placed at the outer canthus of each eye (horizontal EOG) and below and above left eye (vertical EOG). The stimuli were monitored by Presentation (Neurobehavioral Systems, Albany, CA, USA),^4^ and the signal was recorded with IWave (InstEP Systems, Montréal, QC, USA) running on two PCs.

### ERP Extraction from Raw EEG Signal

Ocular artifacts were corrected offline with the Gratton algorithm (62). Raw signals were averaged offline and time-locked to the stimulus onset, in a time window of 100 ms prior to stimulus onset until 900 ms after stimulus onset. Stimuli were categorized across frequent and rare conditions. ERP data were filtered offline with a 0.30-Hz high-pass filter and a 30-Hz low-pass filter. During the averaging procedure, clippings due to amplifiers saturation and remaining epochs exceeding 100 μV were removed. Finally, participants had to have at least 20 valid trials in each condition to be included in the analyses.

The amplitude of the P200 was calculated as the maximum peak during the 150–300 ms interval, whereas the amplitude of the N200 was calculated as the lowest peak during the same interval. The amplitude of the P300 component was calculated as the mean amplitude in the 300–550 ms interval. Thirty electrodes were used to analyze each of these components: AF1, AF2, AF3, AF4, F1, F2, F3, F4, F5, F6 (frontal region), FC1, FC2, FC3, FC4, C1, C2, C3, C4, C5, C6 (central region), CP1, CP2, CP5, CP6, P1, P2, P3, P4, P5, and P6 (parietal region).

### Statistical Analyses

Since the control group was only tested once, two separate sets of analyses were performed. The first set of analyses compared the TD, BFRB, and control groups at the baseline, whereas the second set of analyses compared the TD and BFRB groups at baseline and after CoPs therapy. Therefore, we performed each MANOVA twice, first with the between-group factor group (TD/BFRB/controls), and then the within-group factor therapy (pre/post) was added. The between-group factor Group only contained two levels in this second set of analyses (TD/BFRB). Independent samples *t*-tests were performed to compare the two groups on age, intelligence, depression, and anxiety scores. Paired samples *t*-tests were also performed to compare TSGS, YGTSS, BDI, and BAI scores before and after the therapy.

To compare TD and BFRB patients with controls on N200, P200, and P300 peak amplitude, repeated-measures MANOVAs were performed with the between-group factor Group (TD/BFRB/controls), and three within-group factors: condition (frequent/rare), region (frontal/central/parietal), and hemisphere (left/right). To assess the therapy effects, a within-group factor therapy was added (pre/post) in the second set of analyses. Significant interactions in all components were further analyzed with paired and independent samples *t*-tests. Further analyses were performed on each clinical group (TD and BFRB) to examine if the impact of CoPs therapy differed between groups. Huynh–Feldt corrections for repeated-measures analyses were performed when required. Tukey’s test was used to assess differences between groups before therapy.

## Results

### Impact of CoPs Therapy on Clinical Measures

The therapy induced a reduction in tics and habits symptoms in TD and BFRB patients, respectively. In both groups, there were reductions in TSGS [*F*(1,51) = 67.09, *p* < 0.001] and YGTSS total scores [*F*(1,51) = 89.13, *p* < 0.001]. Reductions in TSGS total score remained significant when covarying for depression [*F*(1,51) = 26.39, *p* < 0.001] and anxiety [*F*(1,51) = 23.99, *p* < 0.001]. With impulsivity as a covariant, there was a trend toward a significant reduction in TSGS score [*F*(1,50) = 3.23, *p* = 0.078]. Reductions in YGTSS total score remained significant when covarying for depression [*F*(1,51) = 31.16, *p* < 0.001], anxiety [*F*(1,51) = 17.07, *p* < 0.001], and impulsivity [*F*(1,50) = 5.15, *p* < 0.05].

There were also reductions in YTGSS tics/habits impairment [*F*(1,51) = 60.42, *p* < 0.001] and motor tics/habits subscales [*F*(1,51) = 55.84, *p* < 0.001]. Moreover, there was a therapy by group interaction on the YGTSS motor tics/habits subscale [*F*(1,51) = 5.84, *p* < 0.05], which showed that motor tics/habits severity decrease following CoPs therapy in both patient groups, but improvements were more pronounced in the BFRB group. Moreover, the therapy induced a significant improvement in YGTSS scores on the phonic tic subscale in TD patients [*F*(1,25) = 19.30, *p* < 0.001], as well as reduced MGH scales scores for BFRB patients [*F*(1,23) = 25.90, *p* < 0.001]. Following therapy, anxiety and depressive symptoms were also diminished in both patient groups, as shown by significant reductions in BAI [*F*(1,51) = 6.29, *p* < 0.05] and BDI scores [*F*(1,51) = 26.69, *p* < 0.001]. The CoPs therapy had no impact on impulsivity. Clinical results are shown in Table 2.

**Table 2:** **CBT impact on clinical scales**.

			Pre				Post							
			TD (*n* = 26)		BFRB (*n* = 27)		TD (*n* = 26)		BFRB (*n* = 27)					
			Mean	SD	Mean	SD	Mean	SD	Mean	SD	*F*	*p*	*d*	Group difference
Depression (BDI)			11	10.2	14	*7.8*	6	6.5	7	*6.0*	26.69***	**<0.001**	0.73	TD and BFRB: pre > post
Anxiety (BAI)			8	5.9	11	*6.6*	6	6.5	8	*4.7*	6.29*	**<0.05**	0.41	TD and BFRB: pre > post
OCS (Padua)^a^			30	30.9	35	*25.8*	28	23.5	35	*24.4*	0.22	ns	0.04	
Tic severity	TSGS total score		18	9.8	17	*9.7*	9	8.6	7	*7.0*	67.09***	**<0.001**	1.06	TD and BFRB: pre > post
	YGTSS	Total	40	15.3	28	*10.8*	26	11.2	16	*9.3*	89.13***	**<0.001**	1.04	TD and BFRB: pre > post
		Tics/habits impairment	20	10.5	14	*5.9*	10	5.0	7	*5.2*	60.42***	**<0.001**	1.11	TD and BFRB: pre > post
		Motor tics/habits severity	13	4.3	13	*3.5*	11	4.6	8	*4.4*	55.84***	**<0.001**	0.86	TD and BFRB: pre > post
		Phonic tics severity^b^	7	5.6	N/A	N/A	5	4.7	N/A	N/A	19.30***	**<0.001**	0.53	TD: pre > post
MGH scales^c^			N/A	N/A	17	*3.6*	N/A	N/A	10	*5.6*	25.90***	**<0.001**	1.49	BFRB: pre > post
Impulsivity (BIS-10)^d^			71	8.8	72	*7.9*	69	9.0	71	*7.4*	2.76	ns	0.13	

*BDI, Beck depression inventory; BAI, Beck anxiety inventory; OCS, obsessive–compulsive symptoms; TSGS, Tourette’s syndrome global scale; YGTSS, Yale Global Tic Severity Scale; MGH scales, Massachusetts General Hospital Hairpulling Scale and its adapted versions for other BFRB; ns, not statistically significant; d, Cohen’s d were calculated with both clinical groups pooled together, except for YGTSS phonic tics subscale (TD only) and MGH scales (BFRB only)*.

*p < 0.05.

***p < 0.001.

*^a^11 TD patients and five BFRB patients with missing data*.

*^b^Only for TD patients*.

*^c^Only for BFRB patients. Three patients with missing data*.

*^d^One TD patient with missing data*.

*Every significant result is in bold*.

### Counting Oddball Task

#### P200 Component

**Figure 1:**
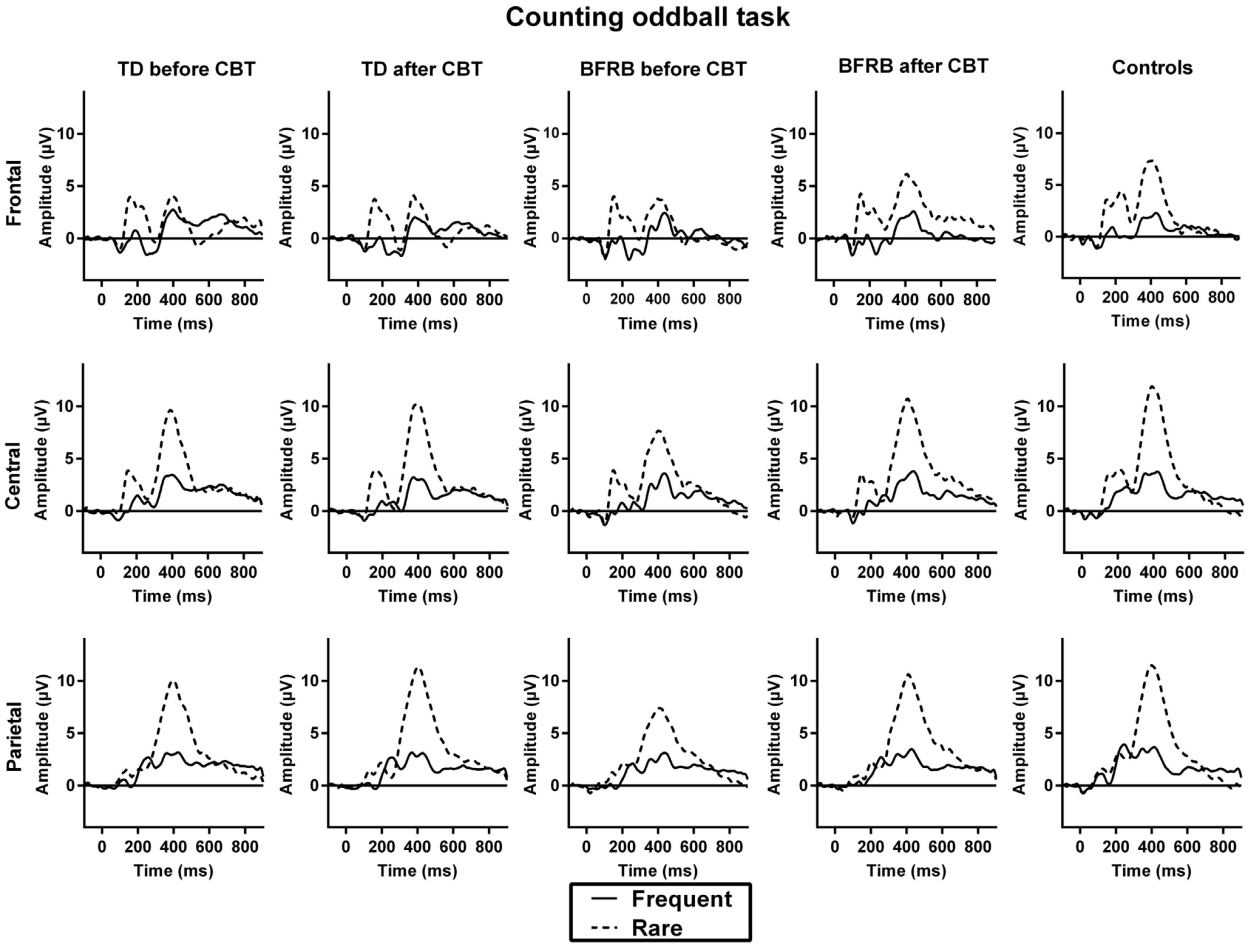
**ERP waveforms during the counting oddball task**. The initial positive deflection that arises about 200 ms after stimulus presentation corresponds to the P200 component. The negative deflection that follows is the N200, which is then followed by the P300, a positive deflection that emerges 300 ms after stimulus presentation. The oddball effect is represented by the P300 amplitude to rare (dotted line) − frequent (solid line) stimuli. Before therapy, TD and BFRB patients had reduced P300 amplitude than controls during rare trials. A significant amplitude increase was induced by the CoPs therapy. This increase occurred in all three regions in BFRB patients but was more localized in the parietal region in TD patients.

Before CoPs therapy, there were main effects of condition [*F*(1,77) = 170.52, *p* < 0.001], region [*F*(2,76) = 7.30, *p* < 0.005], and hemisphere [*F*(1,77) = 15.80, *p* < 0.001]. The rare–frequent oddball effect was larger over the central region in all groups, which lead to a condition by region interaction [*F*(2,76) = 80.50, *p* < 0.001]. There was no group main effect or interaction for that component. No therapy effect reached statistical significance. ERP waveforms for the counting oddball task are shown in Figure 1.

#### N200 Component

Before CoPs therapy, there was a region main effect [*F*(2,76) = 12.71, *p* < 0.001], as well as condition by region [*F*(2,76) = 13.86, *p* < 0.001] and region by hemisphere [*F*(2,76) = 4.58, *p* < 0.05] interactions. There was also a condition by region by hemisphere by group interaction [*F*(3.89,149.63) = 23.65, *p* < 0.05], which revealed that BFRB patients had a larger N200 amplitude than controls over the right-central region during frequent stimuli [*F*(2,77) = 3.36, *p* < 0.05, Tukey: *p* < 0.05], thus reducing the N200 oddball effect. No significant change due to therapy was noted.

#### P300 Component

Before CoPs therapy, there were main effects of condition [*F*(1,77) = 97.94, *p* < 0.001], region [*F*(1.30,100.32) = 51.46, *p* < 0.001], and hemisphere [*F*(1,77) = 4.31, *p* < 0.05], as well as condition by region [*F*(1.34,103.02) = 45.58, *p* < 0.001] and condition by hemisphere [*F*(1,77) = 4.75, *p* < 0.05] interactions.

**Figure 2:**
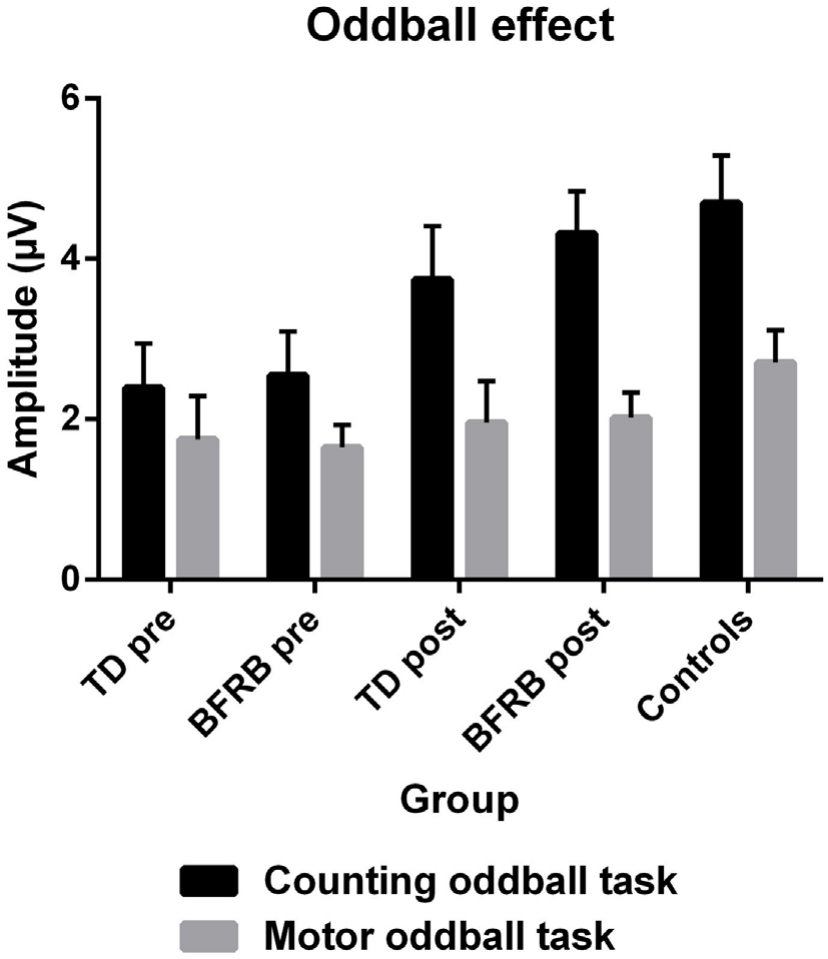
**The P300 oddball effect (therapy by condition)**. The P300 oddball effect represents the subtraction of frequent condition from the rare condition across all scalp regions. With the counting oddball task, the oddball effect was significantly reduced in both clinical groups at pretherapy (black). However, there were no significant differences across groups during the motor task (gray) and no effect of therapy reached significance. At posttherapy, a normalization of the oddball effect was induced during the counting oddball task (black), especially in BFRB patients, where it almost reaches the level of control participants. Note: error bars represent the SEM.

Most importantly, there was a condition by group [*F*(2,77) = 5.26, *p* < 0.01] interaction, which revealed smaller P300 amplitude during rare trials for both clinical groups, in comparison with the control group (Figure 2). This interaction remained significant even when covarying for medication [*F*(2,76) = 4.65, *p* < 0.05]. There was also a condition by region by hemisphere by group four-way interaction [*F*(3.34,128.65) = 3.20, *p* < 0.05], which revealed that there were significant between-group differences during rare trials over the left frontal [*F*(2,77) = 3.25, *p* < 0.05], left [*F*(2,77) = 3.56, *p* < 0.05] and right-central [*F*(2,77) = 3.34, *p* < 0.05], and right parietal [*F*(2,77) = 3.35, *p* < 0.05] regions. There were no such group differences during frequent trials.

When clinical groups were pooled together, the TSGS global score was negatively correlated with the P300 oddball effect in the right-central (*r* = −0.28, *p* < 0.05) and the left (*r* = −0.27, *p* < 0.05) and right (*r* = −0.28, *p* < 0.05) parietal regions. In the TD group, the P300 oddball effect was positively correlated with the BIS-10 score in the left-central (*r* = 0.43, *p* < 0.05) and parietal regions (*r* = 0.48, *p* < 0.05). There was no such correlation in the BFRB or the control group.

**Figure 3:**
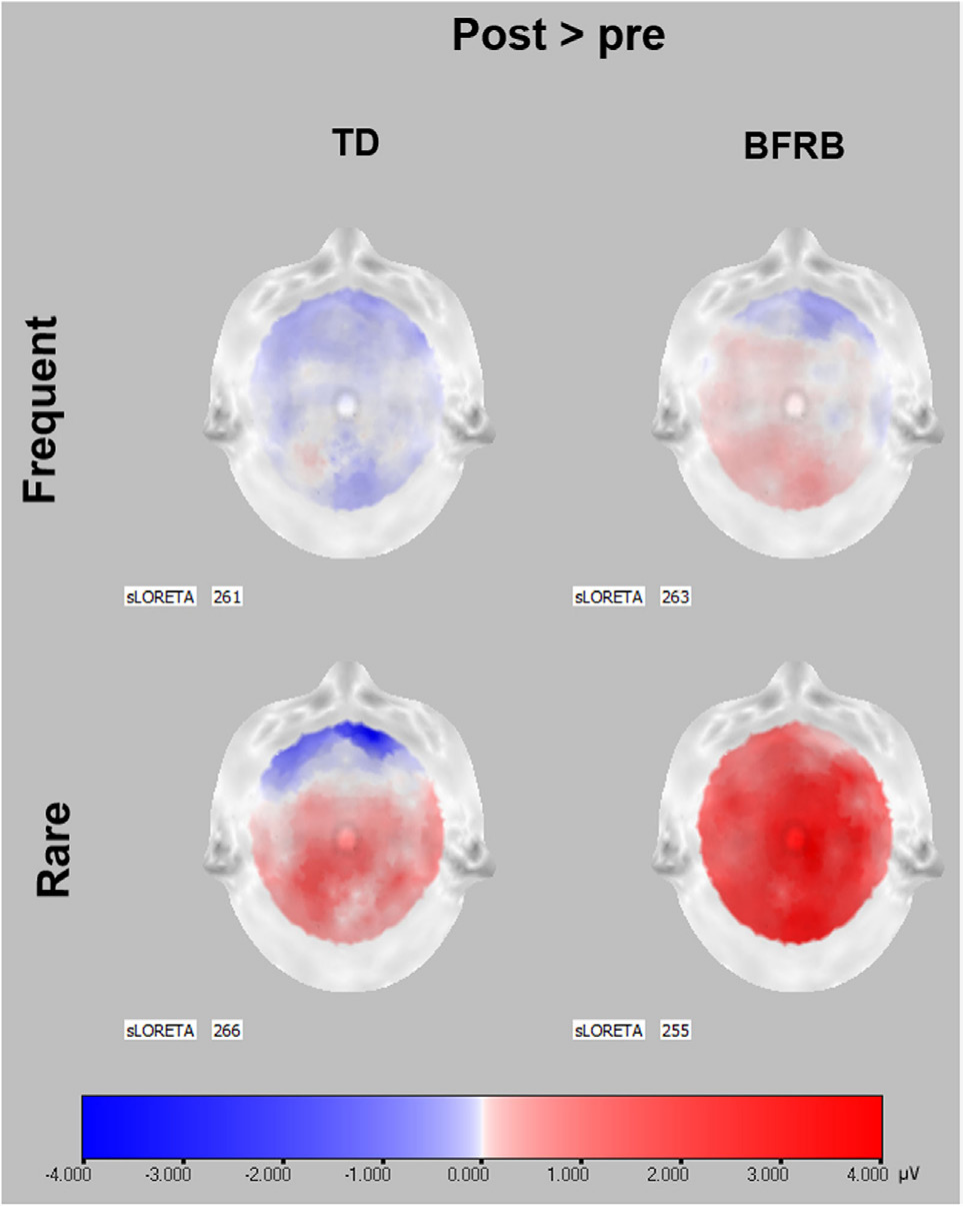
**P300 scalp topographies of activation changes induced by CoPs therapy**. P300 data before therapy were subtracted from P300 data after CoPs therapy to illustrate the activation changes induced by CoPs therapy in frequent and rare conditions. Red color represents an activation increase following CoPs therapy, whereas blue color represents a decrease in activation in microvolts. The SLORETA number indicates the timeframe of each scalp. The timeframes were selected as the maximum peak during the 300–550 ms interval following stimulus presentation, for the frequent and rare condition. For both groups, scalp topographies show that most of the pre–posttherapy difference in P300 activation occurred during rare condition. In TD patients, the activation increase was localized in the parietal area, especially the central and left hemisphere. In BFRB patients, the increase was generalized to the whole cortex. Scalp topographies were obtained through LORETA (63).

There was a main effect of therapy [*F*(1,51) = 5.20, *p* < 0.05], and a therapy by condition interaction [*F*(1,51) = 10.63, *p* < 0.005], which revealed an increase in amplitude during rare trials following therapy (see Figure 2). When covarying with medication, the therapy main effect was no longer significant, but the therapy by condition interaction remained significant [*F*(1,50) = 5.42, *p* < 0.05]. Also, when we analyzed groups separately, there was a therapy main effect [*F*(1,26) = 4.61, *p* < 0.05] and a therapy by condition interaction [*F*(1,26) = 8.17, *p* < 0.01] in the BFRB group (which also revealed amplitude increase in rare trials). In comparison, there was only a trend toward a therapy by condition interaction in the TD group [*F*(1,25) = 3,39, *p* = 0.078], when analyzing the entire cortex. However, there was a localized therapy by condition interaction in the left parietal region [*F*(1,25) = 3.88, *p* < 0.05] in TD patients, revealing an amplitude increase during rare trials and thus, a larger oddball effect in this region after CoPs therapy (Figure 3).

### Motor Oddball Task

#### Reaction Times

Before CoPs therapy, there was a main effect of condition [*F*(1,77) = 169.37, *p* < 0.001], which indicated that all participants responded faster to frequent than to rare stimuli. There was also a group main effect [*F*(2,77) = 4.02, *p* < 0.05] on median reaction times, which revealed that BFRB patients reaction times were delayed compared to the control group (Tukey: *p* < 0.05). There was no significant difference between TD patients and controls and no significant effect of therapy *per se* on reaction times.

#### P200

**Figure 4:**
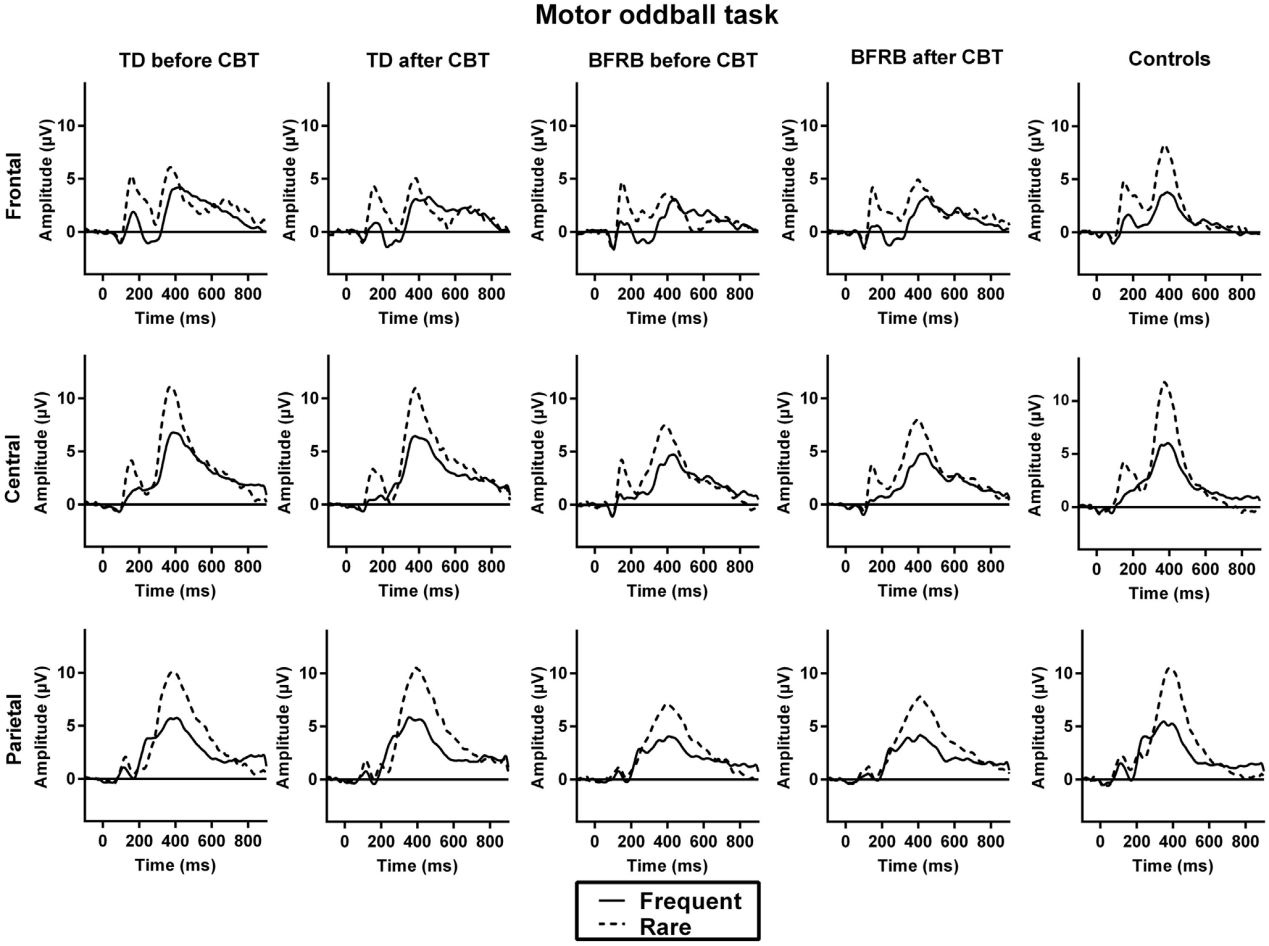
**ERP waveforms during the motor oddball task**. No significant group differences were observed during the motor oddball task.

Event-related potentials waveforms for the motor oddball task are shown in Figure 4. Before CoPs therapy, there were condition by region [*F*(2,76) = 98.10, *p* < 0.001], condition by hemisphere [*F*(1,77) = 16.45, *p* < 0.001], and region by hemisphere [*F*(2,76) = 10.87, *p* < 0.001] interactions.

#### N200

Before CoPs therapy, there were condition by region [*F*(2,76) = 10.44, *p* < 0.001] and condition by hemisphere [*F*(1,77) = 12.62, *p* < 0.01] interactions, which revealed a larger condition effect over the frontal left hemisphere.

#### P300

Before CoPs therapy, there were main effects of condition [*F*(1,77) = 71.57, *p* < 0.001] and region [*F*(2,76) = 41.45, *p* < 0.001] followed by condition by region [*F*(2,76) = 13.65, *p* < 0.001] and condition by hemisphere [*F*(1,77) = 45.81, *p* < 0.001] interactions. There was no significant group difference or effect of therapy in all three components during the motor oddball task (see Figure 3).

## Discussion

The main goal was to compare brain function in TD and BFRB patients during two oddball tasks and to record the effect of the CoPs therapy on clinical measures and brain functioning. To achieve this goal, we used ERP, a technique with high temporal resolution, which is well suited to follow complex stages of the processing stream. We expected that the CoPs therapy would induce a significant reduction in tic symptom severity in both clinical groups, whereas an increase in P300 amplitude was hypothesized to accompany that clinical improvement.

Our results showed that the P300 oddball effect was reduced in both clinical groups. Then, the CoPs therapy induced a normalization of the P300 oddball effect. The clinical change following therapy confirmed our hypothesis with a significant reduction in tics and habit disorders scale scores. Moreover, anxiety and depression symptoms also improved following therapy. These results were observed only in the counting oddball where no motor response was required.

### Counting Oddball Task

Habit symptoms induced an increase in N200 amplitude over the right-central region, during the counting oddball task. Indeed, in BFRB patients, the N200 was larger for frequent stimuli, thus reducing the oddball effect. In an oddball paradigm, the N200 is traditionally representative of attention and detection processes (64). At a functional level, this central N200 is generated by the anterior cingulate cortex and is related to conflict monitoring and cognitive control (64, 65). The observed N200 asymmetry toward the right hemisphere could be caused by the impaired functioning of the corpus callosum (66). The corpus callosum and the prefrontal cortex have a role in mediating interhemispheric interference (67). Smaller corpus callosum could be due to accelerated pruning, whereas axonal pruning is reduced in the frontal cortex of TD patients (68). Therefore, such reports are consistent with our results of hemispheric discrepancy in the frontal and central regions, and the BFRB group seems to share that characteristic with the TD.

Since the N200 reflects monitoring and control, an increase in N200 amplitude could be considered as a function of the amount of effort that the individual put into regulating the urge to perform their habits and/or tics. However, the fact that the therapy failed to affect the N200 oddball effect could mean that despite better tics/habits awareness and modification of action style, this is not reflected by cerebral activity, at least in that ERP temporal window.

Later in the processing stream, for both patient group there was a significant reduction of the P300 oddball effect, particularly over the left anterior hemisphere (frontal and central) and the right posterior hemisphere (central and parietal). Moreover, the P300 oddball effect in the right-central region and bilaterally in the parietal region was negatively correlated with TSGS score, showing that the P300 oddball effect was reduced when tic/habits symptoms were more severe. Such correlation was not found with the YGTSS total score or one of its subscales. This could be explained by the fact that the TSGS has a more detailed behavioral subscale, including individual rating of learning problems, occupational problems, and motor restlessness (52). On the other side, the YGTSS has a 0–50 impairment subscale in which global impairment caused by TD is scored (53). Therefore, this difference between those two scales could explain why we found correlations between the P300 oddball effect with the TSGS, but not with the YGTSS.

The P300, which indexes processes of stimulus evaluation and categorization (69, 70), is generated by a network that includes the prefrontal cortex, the temporoparietal junction, the inferior parietal lobule, the supramarginal gyrus, and the cingulate gyrus (70, 71). In a study on a specific subtype of BFRB (i.e., trichotillomania) with MRI, it was reported that patients show higher levels of gray matter in the cingulate and parietal regions, in comparison with healthy controls (15). Trichotillomania patients also showed impairments in white matter tracts in the anterior cingulate gyrus, as shown by reduced fractional anisotropy in that region (16). In comparison, TD patients showed decrease gray matter in the anterior cingulate gyrus and the sensorimotor areas and reductions in white matter in the right cingulate gyrus (72). The P300 reduction has been related to impairments in gray matter of these regions (73), whereas another study reported positive correlations between P300 amplitude and white matter volumes in the prefrontal cortex and the temporoparietal junction, which were found in both healthy controls and patients at risk for psychosis (74). Therefore, P300 reduction could potentially reflect reduced white or gray matter of the prefrontal cortex and sensorimotor regions of the brain that in turn affect tics/habit symptoms.

Interestingly, the non-motor P300 oddball effect increased in both clinical groups following therapy. While this enhancement was found over the entire cortex in BFRB patients, it was localized to the parietal cortex in TD patients. One component of CoPs treatment model for tics and habits is awareness training, in which patients learn to better integrate information from the social, geographical, physical, and emotional context (12). Hence, the larger P300 oddball effect, found after therapy during a non-motor task, may depict enhance cognitive resources mobilized for working memory and contextual updating processes acquired through persistent training, during the CoPs therapy and practice sessions. Thus, the treatment may promote normalization of aberrant cortical pathways in adults with TD and BFRB. The change in P300 oddball effect could also represent an adaptive mechanism to update information in working memory despite reduced gray and white matter in sensorimotor and prefrontal areas (7, 8, 72, 75). Our findings are also consistent with recent findings in fMRI, which revealed that patients with greater tic severity reduction had higher activity in the inferior frontal gyrus (30). The authors argue that since the inferior frontal gyrus is involved in task-switching and set-shifting, greater activity of this region could be associated with less impairment in TD patients. However, these results were obtained from a motor inhibition priming task, which differ from our own non-motor oddball task that mobilize cerebral structures, such as the cerebellum, the thalamus, and the frontal and parietal cortex (48). Intriguingly, our posttherapy increase was found only with the counting oddball task, which could suggest that the non-motor P300 amplitude forms a good marker of tic/habits normalization that accompanies change in cortical activation.

### Motor Oddball Task

Consistently, our ERP results during the motor oddball task confirmed that there were no significant group difference in all components during the motor oddball task and these ERP components, along with the reaction times, also were not affected by the CoPs therapy. While all participants showed delayed reaction times for rare than for frequent stimuli, which is expected with this type of motor oddball task, both clinical groups’ reaction times were not significantly different from controls. This is consistent with prior findings with similar oddball paradigms in TD patients (39). Intact reaction times in adults with TD have also been found in Go/NoGo motor inhibition tasks (76, 77) and during a stimulus–response compatibility paradigm (13, 78).

As seen in Figure 2, the oddball effect is generally smaller in the motor than the counting task, in all groups. The amplitude of the P300 oddball effect during the motor task does not differ between groups. Motor-related potentials have been reported to overlap with the P300 and, thus, motor responses can have an attenuating effect on P300 component (79, 80). This could explain, in part, why that motor-related P300 was not significantly affected by tic/habit symptoms or by therapy in the motor oddball task. This suggests that TD and BFRB patients do not differ from healthy controls in the evaluation of stimuli salience and its task-related adequacy (N200/P200) in the context of a motor oddball task. Again, this is consistent with prior research on adults with TD that also showed intact P200 in counting oddball paradigm (42).

### Limitations

The principal limitation of the current study is the fact that the control group was only tested once. Ideally, controls could have been tested a second time, with the same time interval between electrophysiological recordings than our patient groups. However, previous investigations showed good test–retest reliability of the P300 amplitude over time (81, 82), suggesting that control participants’ electrocortical activity would not differ significantly in a second recording. Another limitation is that there were more males in the TD group and more females in the BFRB group, but this is consistent with the inherent gender ratio of both disorders (9, 83). Literature on this matter does not reveal significant gender difference on P300 amplitude in oddball paradigms (84–86).

Also, some patients were under medication, and others had sub-clinical comorbid disorders. Even though some of our results could be explained by these factors, we chose to include patients with comorbidities to have a better ecological validity, since comorbidities are the norm rather than the exception in TD (9, 87) and BFRB as well (88, 89). Finally, clinical scales were administered by unblinded clinicians, which could have affected the rating of symptom severity.

## Conclusion

Our findings constitute one of many building blocks that seek integration of psychophysiological measures into evidence-based treatment of TD and BFRB. Consistent with that approach, the CoPs model considers the release of tension as a part of a general regulation system, which postulates that the evaluation of tics must focus further on situational triggers and on a particular style of action characterized by sensorimotor functioning that tends to increase muscular activation and tension. Our results allowed to improve the cerebral and cognitive outcome following the CoPs therapy, for these clinical groups. In conclusion, we demonstrated that TD and BFRB patients have smaller P300 oddball effect, reflecting impairments in attention and working memory. We also found a modification of this neural process after therapy, which was generalized throughout all brain regions in BFRB patients and more localized in the parietal motor area in TD patients.

## Author Contributions

SMB has written this article in partial fulfillment for his doctoral thesis in neuroscience. KO is chief of the Tourette and OCD clinic, and he was responsible for English text revision for the current article. MR performed the analyses and wrote some sections of the manuscript. GS has co-written this article with SMB, particularly the pretherapy phase. JL was responsible, with KO, of the CoPs treatment. She also made editorial revisions. PB was responsible for the differential diagnosis. He also made editorial revisions. ML supervised all aspects of data acquisition and analysis with the first author. He also made editorial revisions.

## Conflict of Interest Statement

The authors declare that the research was conducted in the absence of any commercial or financial relationships that could be construed as a potential conflict of interest.

## Footnotes

^1^Twenty of the 26 TS patients and 19 of the 27 controls included in this study were also included in one of our previous study, but with a different experimental task (13).

^2^Contact the authors for more information about the CoPs program. Also, see Lavoie et al. (25) or O’Connor et al. (26) for further details.

^3^http://www.sandsresearch.com/electrode-caps.html

^4^http://www.neurobs.com/
